# Prevalence and factors associated with atrial fibrillation among adults with type 2 diabetes mellitus at Hoima Regional Referral Hospital, Western Uganda: a cross-sectional study

**DOI:** 10.1186/s40842-026-00327-y

**Published:** 2026-08-09

**Authors:** Abdisamad Guled Hersi, Abdisalam Ahmed Sandeyl, Farah Dubad Abdi, Mohamed Jayte, Venance Emmanuel Mswelo, Zakarie Abdullahi Hussein, Hanan Asad, Hamdi Ahmed Jama, Abdirizak Abdinasir Yusuf, Abdifatah Hersi Karshe, Ezera Agwu, Mutaz Ali Abdallah, Jacinto Amandua, Abishir Mohamud Hirsi

**Affiliations:** 1 Department of Internal Medicine, Faculty of Clinical Medicine and Dentistry, Kampala International University Western Campus, Ishaka, Bushenyi, Uganda; 2 Department of Obstetrics and Gynecology, Faculty of Clinical Medicine and Dentistry, Kampala International University Western Campus, Ishaka, Bushenyi, Uganda; 3 Department of Microbiology and Parasitology, College of Medicine and Health, University of Rwanda, Kigali, Rwanda

**Keywords:** Atrial fibrillation, Type 2 diabetes mellitus, Poor glycemic control, Hypertension, Obesity, HIV infection, Uganda

## Abstract

**Background:**

Atrial fibrillation (Afib) and type 2 DM (T2DM) are common conditions associated with substantial cardiovascular morbidity and mortality. However, data on the burden of Afib among patients with T2DM in sub-Saharan Africa remain limited. This study aimed to determine the prevalence, clinical features, and factors associated with Afib among adults with T2DM at Hoima Regional Referral Hospital (HRRH), western Uganda.

**Methods:**

A hospital-based cross-sectional study was conducted among 355 adults with T2DM attending HRRH. Participants underwent clinical evaluation, glycated hemoglobin (HbA1c) testing, and 12-lead electrocardiography (ECG) for Afib diagnosis. Logistic regression analysis was used to identify factors associated with Afib, with statistical significance set at *p* < 0.05.

**Results:**

The prevalence of Afib was 7.9% (28/355). Patients with Afib commonly presented with palpitations, dizziness, fatigue, chest pain, and dyspnea, with tachycardia and irregular pulse frequently observed on examination. In multivariable analysis, hypertension (aOR 3.027, 95% CI 1.419–6.431; *p* = 0.020), human immunodeficiency virus (HIV) infection (aOR 2.026, 95% CI 1.738–6.538; *p* = 0.048), body mass index (BMI ≥ 25 kg/m²) (aOR 3.014, 95% CI 1.242–7.930; *p* = 0.029), and poor glycemic control (HbA1c ≥ 8%) (aOR 3.813, 95% CI 1.762–8.265; *p* = 0.007) were independently associated with Afib.

**Conclusion:**

Afib is relatively common among adults with T2DM in western Uganda, affecting nearly one in ten patients. Hypertension, HIV infection, obesity, and poor glycemic control were significant associated factors. Integrating ECG screening into diabetes care, particularly for high-risk patients may improve early detection and management in resource-limited settings.

## Introduction

Atrial fibrillation (Afib) is the most common sustained cardiac arrhythmia worldwide and is associated with increased risks of stroke, heart failure, and all-cause mortality. It is characterized by disorganized atrial electrical activity resulting in ineffective atrial contraction, typically presenting as an irregularly irregular pulse and absence of P waves on electrocardiography (ECG) [1].

Type 2 diabetes mellitus (T2DM), is a chronic metabolic disorder characterized by persistent hyperglycemia due to insulin resistance and impaired insulin secretion [2]. Afib and T2DM frequently coexist and share several pathophysiological mechanisms, including systemic inflammation, atrial fibrosis, autonomic dysfunction, and electrical remodeling, all of which increase susceptibility to Afib in individuals with T2DM [3].

The global burden of T2DM continues to rise, with over 500 million adults affected worldwide, and T2DM has been shown to independently increase the risk of Afib by approximately 40% [4, 5]. Poor glycemic control, as reflected by elevated glycated hemoglobin (HbA1c), further increases this risk [6].

In sub-Saharan Africa, the burden of T2DM is increasing rapidly, yet data on its cardiovascular complications, particularly Afib, remain limited [7]. In Tanzania, Kalokola et al. reported an Afib prevalence of 6.1% among patients with T2DM based on ECG screening [8]. However, similar studies remain scarce across the region. In Uganda, the prevalence of T2DM is rising in both urban and rural populations [9], yet routine screening for Afib is rarely performed due to limited access to ECG and low clinical awareness, resulting in potential underdiagnosis of asymptomatic Afib.

Given this limited regional evidence, this study provides the first data from Uganda on the prevalence, clinical features, and associated factors of Afib among adults with T2DM. By identifying context-specific risk factors, including both cardiometabolic factors and HIV infection, this study contributes to a better understanding of Afib risk profiles in sub-Saharan Africa.

Therefore, this study aimed to determine the prevalence, clinical features, and factors associated with Afib among adults with T2DM at HRRH, western Uganda.

## Methods

### Study design and setting

This was a hospital-based cross-sectional study conducted at Hoima Regional Referral Hospital (HRRH), a public teaching hospital located in western Uganda. The hospital serves as the main referral center for the Bunyoro sub-region and hosts a weekly diabetes clinic under the Department of Internal Medicine. The clinic provides care to adult patients with diabetes from both urban and rural communities in the region. Data collection was conducted between April and July 2025.

### Study population and eligibility criteria

The study population consisted of adult patients aged 18 years and above with a confirmed diagnosis of T2DM attending the HRRH diabetes clinic during the study period.

### Inclusion criteria

All consenting adults (≥ 18 years) with confirmed T2DM attending the diabetes clinic during the study period were eligible to participate.

### Exclusion criteria

Patients who were critically ill at the time of assessment: defined as those requiring emergency medical intervention or hemodynamic stabilization, were excluded due to potential ECG interference or inability to provide informed consent. Patients taking anti-arrhythmic medications and those with a prior diagnosis of Afib were also excluded from the study.

### Sample size determination

The sample size was estimated using Daniel’s Formula for Sample Size Determination for prevalence studies [10], Assuming a conservative 30% prevalence of Afib among adults with T2DM, based on a hospital-based study from Romania reporting a prevalence of 41.63% [11], and using a 95% confidence level (Z = 1.96) with a 5% margin of error (d = 0.05), the minimum sample size required was 323 participants. To account for potential non-response and to ensure adequate power for multivariable analysis, a 10% adjustment was added, resulting in a final sample size of 355 participants.

### Sampling procedure

Participants were selected using consecutive sampling. All eligible patients attending the diabetes clinic during the study period were invited to participate until the required sample size of 355 participants was reached. This approach was appropriate in the clinical setting, as it allowed for the inclusion of all accessible and eligible patients in a systematic manner, minimizing selective recruitment by the investigators. However, as a non-probability sampling method, consecutive sampling may introduce selection bias and limits the generalizability of the findings beyond similar hospital-based populations.

## Data collection procedures

### Questionnaire and interviews

A structured and pre-tested questionnaire was used to collect data on socio-demographic characteristics, lifestyle factors (smoking and alcohol consumption), clinical symptoms, treatment history, comorbidities, and complications related to T2DM. The questionnaire was administered in English or Runyoro depending on the participant’s preference. Information on hypertension and HIV status was obtained from participants’ clinical records; hypertension was defined by a documented diagnosis or current use of antihypertensive medications, while HIV status was confirmed by documented serological results or antiretroviral therapy use.

### Physical and clinical examination

All participants underwent a physical examination including measurement of weight, height, and blood pressure. Body mass index (BMI) was calculated as weight in kilograms divided by the square of height in meters (kg/m²). Pulse rate and rhythm were assessed at rest by the principal investigator prior to ECG recording.

### Laboratory tests

Venous blood samples (4 mL) were collected from each participant and analyzed for glycated hemoglobin (HbA1c) using a certified chemistry analyzer. Glycemic control was categorized as good (< 8%) or poor (≥ 8%) according to international diabetes management guidelines.

### Electrocardiography (ECG) assessment

A resting 12-lead electrocardiogram (ECG) was performed for each participant by the principal investigator using standard procedures. All ECG recordings were independently reviewed and interpreted by a physician experienced in electrocardiographic analysis. In cases of uncertainty, tracings were re-evaluated to ensure diagnostic accuracy. Afib was diagnosed based on standard electrocardiographic criteria, defined as an irregularly irregular rhythm with absence of discernible P waves [1].

### Validity and reliability of tools

The questionnaire was reviewed by two experts to assess content validity using the Content Validity Index (CVI), with a score of ≥ 0.80 considered acceptable. A pilot test was conducted among 10 patients attending the same clinic to evaluate clarity and reliability of the questionnaire. Internal consistency was assessed using Cronbach’s alpha, with a value of ≥ 0.75 considered acceptable.

### Data management and statistical analysis

Data were entered into Microsoft Excel and exported to SPSS version 26.0 for analysis. Descriptive statistics were used to summarize participants’ baseline characteristics. The prevalence of Afib was calculated with a 95% confidence interval.

Bivariate analysis using the Chi-square test was conducted to identify variables associated with Afib. Variables with a p-value ≤ 0.20 in the bivariate analysis were considered for inclusion in the multivariable logistic regression model to avoid excluding potential confounders at the screening stage, as recommended in previous methodological studies [12]. Adjusted odds ratios (aOR) with 95% confidence intervals were reported, and statistical significance was set at *p* < 0.05. Multicollinearity among independent variables was assessed using the Variance Inflation Factor (VIF), with a threshold of < 5 indicating no significant multicollinearity. Model fitness was evaluated using the Hosmer–Lemeshow goodness-of-fit test.

### Ethical considerations

Ethical approval was obtained from the Kampala International University Research Ethics Committee. Additional administrative clearance was granted by the HRRH administration. Written informed consent was obtained from all participants prior to enrollment in the study. Confidentiality was maintained through the use of coded identifiers, and no personal identifiers were included in the data collection tools. ECG and HbA1c testing were provided to participants at no cost. Participants were compensated with 2,000 Ugandan shillings to cover transport and time. All data collection procedures were conducted in accordance with standard ethical guidelines.

## Results

### Characteristics of the study participants

A total of 355 participants were included in this study. Slightly more than half (54.6%) were male, and 25.6% were aged below 45 years. About 35.2% had lived with T2DM for more than 10 years. The majority (69.0%) were receiving oral antidiabetic medications. Detailed participant characteristics are presented in Table 1.

**Table 1:** Characteristics of study participants

Characteristic	Frequency	Percentage
**Sex**		
Female	161	45.4
Male	194	54.6
**Residence**		
Urban	148	41.7
Rural	207	58.3
**Age category**		
< 45	91	25.6
46–59	142	40.0
60+	122	34.4
**Marital status**		
Single	49	13.8
Married	238	67.0
Divorced	5	1.4
Separated	26	7.3
Widowed	37	10.4
**Religion**		
Protestant	82	23.1
Catholic	200	56.3
Muslim	22	6.2
Other	51	14.4
**Education level**		
None	18	5.1
Primary	242	68.2
Secondary	86	24.2
Tertiary	9	2.5
**Occupation**		
Peasant	220	62.0
Formal employment	41	11.5
Business	71	20.0
Other	23	6.5
**Hypertension**		
No	150	42.3
Yes	205	57.7
**HIV**		
No	310	87.3
Yes	45	12.7
**Smoking**		
No	290	81.7
Yes	65	18.3
**Alcohol intake**		
No	224	63.1
Yes	131	36.9
**Duration of T2DM**		
< 10	230	64.8
10+	125	35.2
**T2DM complication**		
None	216	60.8
Nephropathy	85	23.9
Retinopathy	15	4.2
Neuropathy	29	8.2
Other	10	2.8
**History of CVA**		
No	323	91.0
Yes	32	9.0
**Treatment type**		
Oral	245	69.0
Injectable	9	2.5
Both	83	23.4
Lifestyle modification	18	5.1
**BMI**		
Normal	139	39.2
< 18.5	18	5.1
25+	198	55.8
**HbA1c category**		
< 8	157	44.2
8+	198	55.8

HIV=Human immunodeficiency virus, T2DM=Type 2 Diabetes mellitus, CVA=Cerebrovascular accident, BMI=Body mass index, HbA1c=Glycated hemoglobin

### Prevalence of Afib among adults with T2DM

**Fig. 1:**
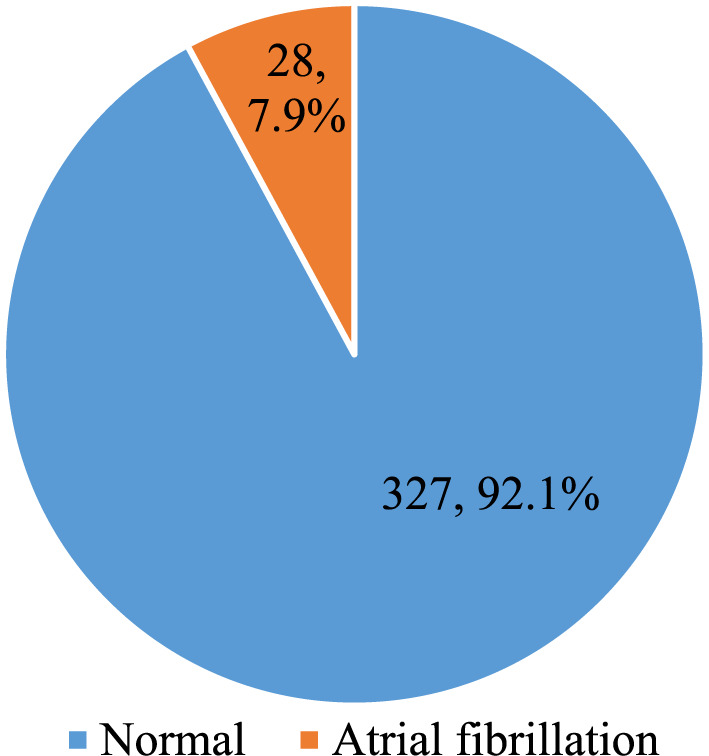
Prevalence of Afib among adults with T2DM

Among the 355 participants, 28 were diagnosed with Afib, giving a prevalence of 7.9% (95% CI 5.4–10.7). The prevalence of Afib among the study participants is illustrated in Fig. 1.

### Clinical features of Afib among adults with T2DM

Palpitations, dizziness, fatigue, chest pain, and difficulty in breathing were significantly more common among participants with Afib (*p* < 0.05 for all). Tachycardia and an irregular pulse were also more frequently observed in this group. Detailed findings are presented in Table 2.

**Table 2:** Clinical features of Afib among adults with T2DM

Clinical feature	Overall	No Afib, *N* = 327	Afib, *N* = 28	*P* value
**Palpitations**				0.036
No	85(23.9)	83(25.4)	2(7.1)	
Yes	270(76.1)	244(74.6)	26(92.9)	
**Dizziness**				**0.015**
No	77(21.7)	76(23.2)	1(3.6)	
Yes	278(78.3)	251(76.8)	27(96.4)	
**Fatigue**				**0.015**
No	74(20.8)	73(22.3)	1(3.6)	
Yes	281(79.2)	254(77.7)	27(96.4)	
**Chest pain**				**0.023**
No	87(24.5)	85(26.0)	2(7.1)	
Yes	268(75.5)	242(74.0)	26(92.9)	
**Difficulty in breathing**				**0.009**
No	201(56.6)	192(58.7)	9(32.1)	
Yes	154(43.4)	135(41.3)	19(67.9)	
**Pulse rate**				**< 0.001**
< 100	239(67.3)	229(70.0)	10(35.7)	
100+	116(32.7)	98(30.0)	18(64.3)	
**Pulse character**				**< 0.001**
Regular	334(94.1)	325(99.4)	9(32.1)	
Irregular	21(5.9)	2(0.6)	19(67.9)	

### Factors associated with Afib among adults with T2DM

Variables with *p* ≤ 0.20 in the bivariable analysis were included in the multivariable logistic regression model. These included sex, age category, hypertension, HIV infection, smoking, alcohol intake, diabetic complications, type of diabetes treatment, body mass index (BMI), and HbA1c category. Detailed results are shown in Table 3.

**Table 3:** Bivariable analysis of factors associated with Afib among adults with T2DM

Characteristic	No Afib, *N* = 327	Afib, *N* = 28	Bivariable analysis		
			cOR	95% CI	*P* value
**Sex**					
Female	152(46.5)	9(32.1)	Ref		
Male	175(53.5)	19(67.9)	1.834	0.806–4.173	**0.148**
**Residence**					
Urban	133(40.7)	15(53.6)	Ref		
Rural	194(59.3)	13(46.4)	0.594	0.274–1.289	0.288
**Age category**					
< 45	87(26.6)	4(14.3)	Ref		
46–59	131(40.1)	11(39.3)	1.826	0.563–5.920	0.315
60+	109(33.3)	13(46.4)	2.594	0.817–8.238	**0.106**
**Hypertension**					
No	145(44.3)	5(17.9)	Ref		
Yes	182(55.7)	23(82.1)	3.665	1.360–9.877	**0.010**
**HIV**					
No	289(88.4)	21(75.0)	Ref		
Yes	38(11.6)	7(25.0)	2.535	1.010–6.360	**0.047**
**Smoking**					
No	271(82.9)	19(67.9)	Ref		
Yes	56(17.1)	9(32.1)	2.292	0.986–5.329	**0.054**
**Alcohol intake**					
No	214(65.4)	10(35.7)	Ref		
Yes	113(34.6)	18(64.3)	3.409	1.523–7.632	**0.003**
**Duration of T2DM**					
< 10	211(64.5)	19(67.9)	Ref		
10+	116(35.5)	9(32.1)	0.862	0.378–1.966	0.723
**T2DM complication**					
None	208(63.6)	8(28.6)	Ref		
Nephropathy	77(23.5)	8(28.6)	2.701	0.980–7.448	**0.055**
Retinopathy	14(4.3)	1(3.6)	1.857	0.217–15.914	0.572
Neuropathy	21(6.4)	8(28.6)	3.905	1.371–9.104	**< 0.001**
Other	7(2.1)	3(10.7)	2.143	1.423–7.242	**0.002**
**History of CVA**					
No	299(91.4)	24(85.7)	Ref		
Yes	28(8.6)	4(14.3)	1.780	0.577–5.494	0.316
**Treatment type**					
Oral	233(71.3)	12(42.9)	Ref		
Injectable	8(2.4)	1(3.6)	2.427	0.280–21.010	0.421
Both	70(21.4)	13(46.4)	3.606	1.574–8.260	**0.002**
Lifestyle modification	16(4.9)	2(7.1)	2.427	0.500-11.787	0.271
**BMI**					
Normal	134(41.0)	5(17.9)	Ref		
< 18.5	16(4.9)	2(7.1)	3.350	0.600-18.705	**0.168**
25+	177(54.1)	21(75.0)	3.180	1.169–8.650	**0.023**
**HbA1c category**					
< 8	152(46.5)	5(17.9)	Ref		
8+	175(53.5)	23(82.1)	3.995	1.483–10.766	**0.006**

cOR= Crude odds ratio, CI= Confidence interval, HIV=Human immunodeficiency virus, T2DM= Type 2 Diabetes mellitus, CVA=Cerebrovascular accident, BMI=Body mass index, HbA1c=Glycated hemoglobin

In the multivariable logistic regression analysis, hypertension (aOR 3.027, 95% CI 1.419–6.431; *p* = 0.020), HIV infection (aOR 2.026, 95% CI 1.738–6.538; *p* = 0.048), BMI ≥ 25 kg/m² (aOR 3.014, 95% CI 1.242–7.930; *p* = 0.029), HbA1c ≥ 8% (aOR 3.813, 95% CI 1.762–8.265; *p* = 0.007) were independently associated with Afib. Detailed results are presented in Table 4.

No evidence of multicollinearity was observed among variables included in the multivariable model, with all Variance Inflation Factor (VIF) values below 5 (range: 1.012–1.036). The Hosmer–Lemeshow goodness-of-fit test indicated that the model was well calibrated (χ² = 6.046, df = 8, *p* = 0.642).

**Table 4:** Multivariable analysis of factors associated with Afib among adults with T2DM

Characteristic	Bivariable analysis			Multivariable analysis		
	cOR	95% CI	*P* value	aOR	95% CI	*P* value
**Sex**						
Female	Ref					
Male	1.834	0.806–4.173	0.148	1.928	0.664–5.595	0.227
**Age category**						
< 45	Ref					
46–59	1.826	0.563–5.920	0.315	1.065	0.217–5.234	0.938
60+	2.594	0.817–8.238	0.106	1.276	0.267–6.090	0.760
**Hypertension**						
No	Ref					
**Yes**	**3.665**	**1.360–9.877**	**0.010**	**3.027**	**1.419–6.431**	**0.020**
**HIV**						
No	Ref					
**Yes**	**2.535**	**1.010–6.360**	**0.047**	**2.026**	**1.738–6.538**	**0.048**
**Smoking**						
No	Ref					
Yes	2.292	0.986–5.329	0.054	1.252	0.378–4.152	0.713
**Alcohol intake**						
No	Ref					
Yes	3.409	1.523–7.632	0.003	1.710	0.534–5.480	0.366
**T2DM complication**						
None	Ref					
Nephropathy	2.701	0.980–7.448	0.055	1.252	0.378–4.152	0.713
Retinopathy	1.857	0.217–15.914	0.572	1.710	0.534–5.480	0.366
Neuropathy	3.905	1.371–9.104	< 0.001	2.427	0.500-11.787	0.271
Other	2.143	1.423–7.242	0.002	1.826	0.563–5.920	0.315
**Treatment type**						
Oral	Ref					
Injectable	2.427	0.280–21.010	0.421	2.319	0.163–7.317	0.381
Both	3.606	1.574–8.260	0.002	2.183	0.806–5.913	0.125
Lifestyle modification	2.427	0.500-11.787	0.271	1.692	0.244–6.726	0.129
**BMI**						
Normal	Ref					
< 18.5	3.350	0.600-18.705	0.168	2.632	0.416–16.639	0.304
**25+**	**3.180**	**1.169–8.650**	**0.023**	**3.014**	**1.242–7.930**	**0.029**
**HbA1c category**						
< 8	Ref					
**8+**	**3.995**	**1.483–10.766**	**0.006**	**3.813**	**1.762–8.265**	**0.007**

cOR= Crude odds ratio, aOR= Adjusted odds ratio, CI= Confidence interval, HIV=Human immunodeficiency virus, T2DM= Type 2 Diabetes mellitus, CVA=Cerebrovascular accident, BMI=Body mass index, HbA1c=Glycated hemoglobin

## Discussion

### Prevalence of Afib

In this study, the prevalence of Afib among adults with T2DM at HRRH, western Uganda was 7.9%. This is comparable to the global pooled prevalence of approximately 7.6% reported in a recent systematic review of multiple T2DM cohorts [13]. Specifically, our prevalence is slightly higher than that reported in a hospital-based cohort in Tanzania, where Afib was observed in 6.1% of T2DM patients [8], and higher than estimates from a Japanese study documenting Afib in 4.4% of adult T2DM patients [14].

Conversely, our prevalence is lower than that reported in a cross-sectional study of older adults with T2DM in Pakistan, where 11.7% of participants had Afib [15]. In the United Kingdom, the overall prevalence of Afib among adults with T2DM was approximately 5.0% in 2016, though it reached 9.9% among patients aged ≥ 75 years [16]. These differences likely reflect variations in age distribution, study settings, burden of cardiovascular comorbidities, and Afib detection strategies, particularly the use of systematic ECG screening.

Taken together, these findings suggest that Afib represents a substantial cardiovascular complication among adults with T2DM in western Uganda, with a prevalence broadly consistent with global estimates. Importantly, unlike prior studies in sub-Saharan Africa such as the Tanzanian cohort, which identified only limited associated factors, this study provides both prevalence data and a broader characterization of associated risk factors, offering a more comprehensive understanding of Afib in this population. In resource-limited settings where routine cardiac screening is uncommon, integrating ECG screening into diabetic care may improve early detection of Afib.

### Clinical features associated with Afib

The predominance of symptoms such as palpitations, shortness of breath, fatigue, chest pain, and dizziness reflects the hemodynamic consequences of irregular ventricular response and impaired cardiac output in Afib. These findings are consistent with previous clinical studies showing that palpitations are among the hallmark symptoms of Afib, reflecting the irregular and often rapid atrial activity [17, 18].

Shortness of breath and fatigue frequently occur due to reduced cardiac output and impaired ventricular filling, while chest pain may result from rapid ventricular rates or underlying ischemic heart disease [19, 20]. Dizziness and light-headedness are also well-described symptoms, arising from reduced cerebral perfusion during episodes of irregular ventricular response [17, 21].

These findings suggest that while symptom-based screening may support early identification in low-resource settings, reliance on symptoms alone may miss asymptomatic or paroxysmal Afib, underscoring the importance of ECG confirmation in high-risk populations.

### Factors associated with Afib

Beyond clinical presentation, identifying associated factors is critical for risk stratification and targeted screening. In this study, hypertension, poor glycemic control (HbA1c ≥ 8%), elevated body mass index (BMI ≥ 25 kg/m²), and HIV infection were independently associated with Afib among adults with T2DM. These findings provide a broader characterization of Afib risk factors in sub-Saharan Africa, extending beyond the limited associations reported in previous regional studies.

Hypertension was independently associated with Afib, consistent with findings from cohort studies conducted in Norway, China, and South Korea, where elevated blood pressure contributed to atrial structural remodeling and increased arrhythmia risk [22–24]. Chronic pressure overload can lead to left atrial enlargement, fibrosis, and electrical instability, which predispose individuals with T2DM to the development of Afib [25].

Poor glycemic control, reflected by elevated HbA1c levels, was also significantly associated with Afib, consistent with findings from studies conducted in Sweden, Japan and the United States, showing that higher HbA1c levels increase the risk Afib among patients with T2DM [26–28]. Chronic hyperglycemia promotes oxidative stress, inflammation, and structural remodeling of atrial tissue, creating a substrate for arrhythmia development [29]. These findings highlight the importance of optimizing glycemic control in patients with T2DM to potentially reduce the risk of Afib.

Higher BMI was another independent associated factor of Afib in this study. This finding is consistent with evidence from a large population-based study conducted in Korea and the United Kingdom, as well as a study from Germany, which reported an increased risk of Afib among overweight and obese individuals with T2DM [30, 31]. Obesity contributes to left atrial enlargement, systemic inflammation, and neurohormonal activation, all of which facilitate the development of Afib [32].

HIV infection was also associated with Afib in our cohort. Similar associations have been reported in studies conducted in the United States, Nigeria, and in a global systematic review of people living with HIV [33–35]. The relationship between HIV and Afib is likely multifactorial, involving chronic immune activation, systemic inflammation, and endothelial dysfunction, which contribute to atrial remodeling and increased susceptibility to arrhythmias, while prolonged survival on antiretroviral therapy has further amplified cardiovascular risk [36].

In regions with high HIV prevalence, such as sub-Saharan Africa, this association has important public health implications. As people living with HIV experience increased longevity, the burden of non-communicable diseases, including Afib and its complications such as stroke and heart failure, is expected to rise. These findings highlight the need for integrated care models that address both infectious and cardiometabolic conditions and suggest that HIV infection should be considered in risk stratification and screening strategies for Afib among patients with T2DM.

While routine ECG screening in diabetes clinics may improve early detection of Afib, its feasibility in resource-limited settings may be limited by cost, infrastructure, and workforce constraints. A more practical approach may involve targeted screening of high-risk patients, particularly those with hypertension, poor glycemic control, obesity, or HIV infection, to optimize resource utilization and maximize clinical benefit.

In interpreting these findings, several methodological considerations should be acknowledged. First, the cross-sectional design limits causal inference, and the possibility of reverse causality cannot be excluded. While poor glycemic control may increase the risk of Afib, it is also possible that Afib, through stress-related catecholamine release and hemodynamic effects, may contribute to worsening glycemic control.

Second, the hospital-based nature of the study introduces potential selection bias, as patients attending tertiary care are more likely to have multiple comorbidities and may not be representative of the general diabetes population. This could lead to an overestimation of the observed associations.

Third, survivorship bias may have influenced the findings, as patients with more severe disease or complications may not survive long enough to be included in outpatient-based studies, potentially leading to an underestimation of the true burden and severity of Afib among patients with T2DM.

### Strengths and limitations

To the best of our knowledge, this is the first study in Uganda evaluating the prevalence, clinical features, and associated factors of Afib among adults with T2DM using ECG confirmation and HbA1c assessment. The use of objective diagnostic tools strengthens the reliability of the findings and provides clinically relevant data to guide clinical practice in diabetes care. In addition, this study addresses an underrepresented population and contributes important evidence to the limited regional data on Afib among patients with T2DM in sub-Saharan Africa.

However, several limitations should be considered. The cross-sectional design precludes establishing causal relationships between the identified risk factors and Afib. In addition, the single-center, hospital-based nature of the study may limit generalizability and introduce potential selection bias, as patients attending the clinic may differ from the general population. Survivorship bias may also have occurred, as individuals with more severe disease may not have survived or presented to the clinic. Afib diagnosis in this study was based on a single resting 12-lead ECG, which may have underestimated the true prevalence due to missed paroxysmal Afib episodes; as a result, the reported prevalence likely reflects point prevalence rather than the cumulative burden of Afib in this population, and the actual prevalence may be higher. The sample size calculation was based on an assumed Afib prevalence of 30% derived from a hospital-based study outside sub-Saharan Africa, which may not accurately reflect the local context; however, this likely resulted in a larger-than-required sample size and improved the precision of the study estimates. Furthermore, echocardiographic evaluation was not performed, limiting the assessment of structural cardiac abnormalities such as left atrial enlargement or valvular heart disease that may contribute to Afib. Despite these limitations, the study provides an important foundation for future multicenter and prospective research in this population.

## Conclusion

The prevalence of Afib among adults with T2DM at HRRH was 7.9%. Patients with Afib commonly reported symptoms including palpitations, dizziness, fatigue, and chest pain. Hypertension, HIV infection, BMI ≥ 25 kg/m², and poor glycemic control (HbA1c ≥ 8%) were identified as independent associated factors of Afib. These findings highlight the importance of targeted screening and comprehensive risk-factor management in diabetes care to improve early detection and prevention of Afib.

### Recommendations

1. Routine ECG screening for Afib should be incorporated into diabetes clinics, particularly for patients with coexisting hypertension, HIV infection, or overweight and obesity.
2. T2DM patients should receive counseling and regular follow-up aimed at achieving optimal glycemic control as a potential strategy to reduce the risk of Afib.
3. Health systems should strengthen integrated chronic disease care models that address both non-communicable diseases and infectious conditions such as HIV.
4. Further multicenter, prospective studies incorporating echocardiographic assessment and long-term rhythm monitoring are needed to better understand the causal relationships and guide national screening strategies.

## Data Availability

The datasets generated and/or analyzed during the current study are available from the corresponding author on reasonable request.

## Abbreviations

Afib: Atrial fibrillation
T2DM: Type 2 diabetes mellitus
HRRH: Hoima Regional Referral Hospital
HbA1c: Glycated hemoglobin
ECG: Electrocardiography
BMI: Body mass index
HIV: Human Immunodeficiency Virus
